# Equity of resource flows for reproductive, maternal, newborn, and child health: are those most in need being left behind?

**DOI:** 10.1136/bmj.m305

**Published:** 2020-02-03

**Authors:** Melisa Martinez-Alvarez, Frederik Federspiel, Neha S Singh, Marco Schäferhoff, Miriam Lewis Sabin, Chima Onoka, Sandra Mounier-Jack, Josephine Borghi, Catherine Pitt

**Affiliations:** 1 MRC Unit in The Gambia at the London School of Hygiene and Tropical Medicine, The****Gambia; 2 Department of Global Health and Development, London School of Hygiene and Tropical Medicine, London UK; 3 Open Consultants, Berlin, Germany; 4 Partnership for Maternal Newborn and Child Health, Geneva, Switzerland; 5 Department of Community Medicine, College of Medicine, University of Nigeria, Enugu, Nigeria

## Abstract

Although equity has improved in recent years, donors and country governments still need to improve the amount and targeting of funding for reproductive, maternal, and child health, say **Melisa Martinez-Alvarez and colleagues**

**Key messages**

- Since 2002 the distribution of external funding to reproductive, maternal, newborn, and child health (RMNCH) has become more equitable and better targeted at the poorest countries and those experiencing the highest mortality
- The aid envelope is not large enough or well enough concentrated to close gaps in domestic government funding between the poorest and middle income countries
- Donors and governments of low and middle income countries should increase their investments for RMNCH
- Donors should further concentrate their funds on the poorest countries and those with the highest maternal, newborn, and child mortality
- Investment is also needed to close serious data and methodological gaps for assessing equity of financing between and within countries

Financing is a crucial determinant of whether all women, children, and adolescents receive the care they need without risk of impoverishment—the goal of universal health coverage. To achieve the aim of leaving no one behind, as set out in the sustainable development goals, both financing (how resources are raised) and funding (how resources are distributed) must be equitable—that is, raised according to ability to pay and distributed according to need.

Although equity of health financing can be assessed at global, national, and subnational levels, most studies of equity have been conducted at the national and subnational levels because financing policy is considered the domain of national governments. Existing studies have tracked donor funding for specific dimensions of women’s, children’s, and adolescents’ health (particularly reproductive, maternal, newborn, and child health (RMNCH)^1^ ^2^ ^3^ ^4^ ^5^); however, few have investigated equity of donor funds. Analyses of domestic funding for RMNCH have been limited by fragmented and scarce data.^6^ ^7^ Yet, anticipated decreases in donor financing and increased emphasis on mobilising domestic health financing^8^ may have important equity implications, making assessment of equity of resources increasingly urgent at global, national, and subnational levels.

## How do we define equitable resource flows?

Equity is a core part of the universal health coverage agenda, but little guidance exists on what equitable funding for RMNCH resembles or how it should be assessed.

In our analysis (box 1) we identify four dimensions of equity, defined by the type of resource flow and the type of equity. We distinguish between two types of resource flows: financial contributions (who pays) and funding allocation (who benefits).^9^ Regarding equity, we distinguish between horizontal equity (equal treatment for equal need or ability to pay) and vertical equity (unequal treatment for unequal need or ability to pay).^13^ A situation may therefore be considered equitable when

Box 1 **Assessment methods**

We use two databases to analyse equity of resource flows between and within countries (see web supplement). To assess donor flows across 150 aid recipient countries from 2002 to 2017, we use the Muskoka2 approach^4^ applied to the creditor reporting system (CRS) of the Organisation for Economic Cooperation and Development.^9^ To assess domestic government and external expenditure, we analyse the World Health Organization’s Global Health Expenditure Database (GHED).^10^ Data specific to aspects of RMNCH were available for only a limited number of countries (range 9 to 39) and years (2010 to 2016), and so we restrict our analysis to data on reproductive health (including maternal and newborn health and family planning) and children under 5 in 2016.

Available financing data did not allow for a financing incidence analysis, a method typically used to assess equity of financial contributions,^11^ or analysis of out-of-pocket financing. We therefore instead assess equity of RMNCH financing between countries by comparing trends in the total amount of donor financing for RMNCH between 2002 and 2017, using Muskoka2 data. We also used the GHED to compare domestic government RMNCH contributions in 2016 between countries of similar and different income groups. To assess equity of financing within countries, we compare the relative contributions of external and domestic government financing of RMNCH using GHED data.

To analyse equity of funding allocation, we compare donor and domestic government funding to metrics of need. We assess funding and need at the country level because available data did not allow assessment at the individual level or by sub-national geographical area. We measure need as income (GDP per capita) and neonatal and under 5 death rates. We use Muskoka2 data to plot concentration curves and calculate concentration indices^12^ for donor funding for the years 2002, 2010, and 2017. To analyse the distribution of combined donor and domestic government RMNCH funding according to need, we use GHED data for 2016, graphed as bar charts (comparing funding by income group) and scatter plots (comparing funding by neonatal and child mortality).

- Those who have similar ability to pay contribute equally (horizontally equitable financing)
- Those with greater ability to pay contribute more (vertically equitable financing)
- Those with equal need benefit equally from RMNCH funding (horizontally equitable funding), and
- Those with greater need receive more funding (vertically equitable funding).

Any given situation may be considered equitable along all, none, or some of these dimensions. We do not consider other concerns regarding funding, including efficiency, country ownership, predictability, sustainability, aid dependency, or fungibility (whether governments reduce domestic health financing in response to external funding).^14^ ^15^ ^16^

We consider three financing sources: out of pocket, domestic government, and external contributions. Out-of-pocket contributions from households are considered least equitable because they represent a higher share of income for the poor.^17^ Domestic government contributions, mainly from taxation, are considered more equitable than out-of-pocket expenditures as these usually require the richer to pay more (although the degree of equity depends on the taxation system and who contributes to taxes).^18^ External funding, which comprises a sizeable part of revenue in low and middle income countries (28% of health funding to low income countries in 2016^10^), originates primarily from taxation and voluntary contributions from residents of (predominantly) high income countries. It is therefore equitable from a global perspective as it redistributes resources from richer to poorer populations. 

## Progress in equity of who finances services

Using the Muskoka2 method we estimate that donor aid for RMNCH increased by 42% between 2010 and 2017 among donors that reported data for both years (average annual rates of increase, 5.1%).^4^ This represents an improvement in vertical equity at a global level. However, while these increases substantially outpaced increases in overall aid, they were similar to increases in aid for the health sector, suggesting that prioritisation of RMNCH within the health sector has remained constant.^4^

**Fig 1:**
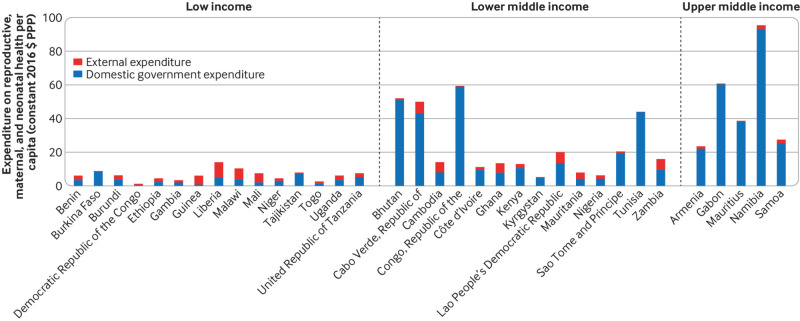
Domestic government and external expenditure on reproductive, maternal, and neonatal health per capita in 2016 (constant 2016 $ PPP), separated by country income group (World Bank 2017 groupings.^19^ Data from the Global Health Expenditure Database^10^

**Fig 2:**
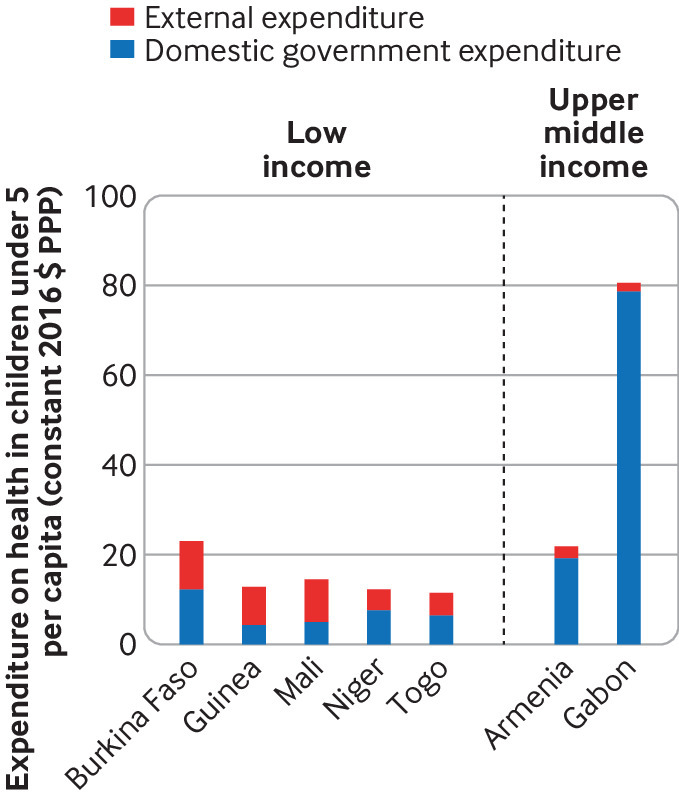
Domestic government and external expenditure on child health per capita in 2016 (constant 2016 $ PPP), separated by country income group (World Bank 2017 groupings^19^). Data from the Global Health Expenditure Database^10^

Our assessment was limited by the small number of data points within the Global Health Expenditure Database (GHED). As expected, per capita domestic government expenditures were higher in (comparatively) richer countries than in poorer countries (fig 1). Contributions from countries of similar income, however, varied substantially. For example, across 14 low income countries, domestic government expenditure on reproductive health in 2016 varied from $0.2 (constant 2016 purchasing power parity, PPP) per capita in the Democratic Republic of the Congo to $8.2 in Burkina Faso. Similarly, across 17 lower middle income countries, domestic government expenditure on reproductive health in 2016 varied from $3.9 per capita in Nigeria to $59 in the Republic of the Congo (fig 1). Domestic financing of reproductive health exceeded donor financing in nine of the 15 low income countries and in all 14 lower middle income and five upper middle income countries, while domestic financing of child health exceeded donor financing in three of five low income countries and both upper middle income countries for which data were available (fig 2).

## Progress in equity of who benefits

**Fig 3:**
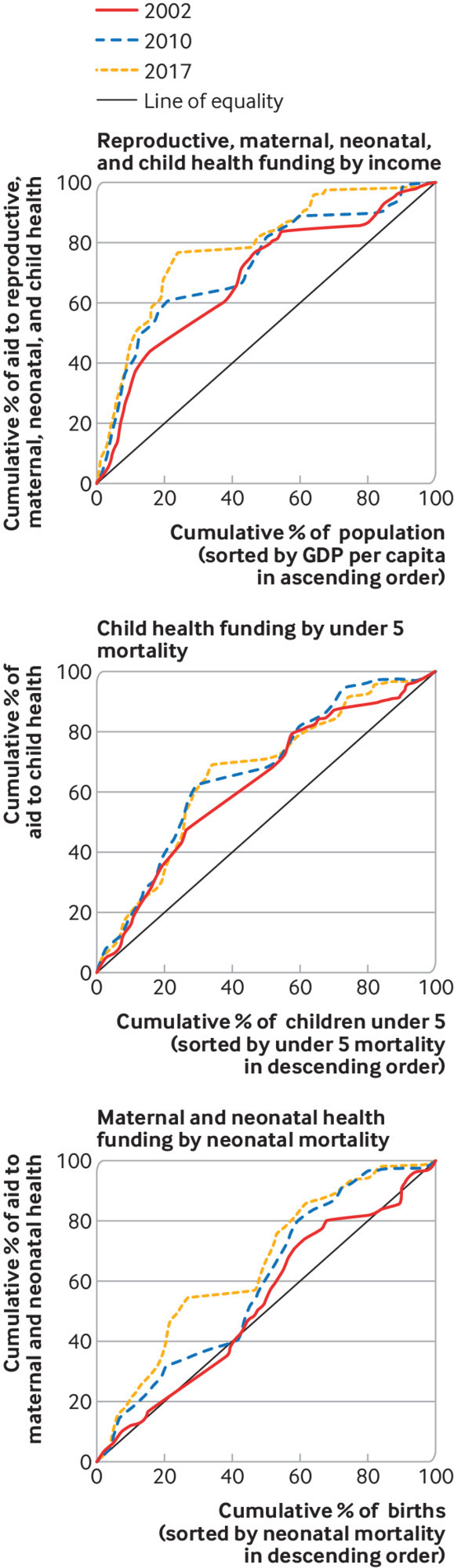
Vertical equity of donor funds for reproductive, maternal, neonatal and child health (RMNCH) between 2002 and 2017.^4^ Concentration curves showing the alignment of global aid to RMNCH with different measures of need in 2002, 2010, and 2017 (only countries with available data are shown). If the curve is above the (diagonal) line of equality, funding is concentrated among those with greater need, whereas if the curve falls below the line of equality, the funding distribution favours those with lesser need

**Fig 4:**
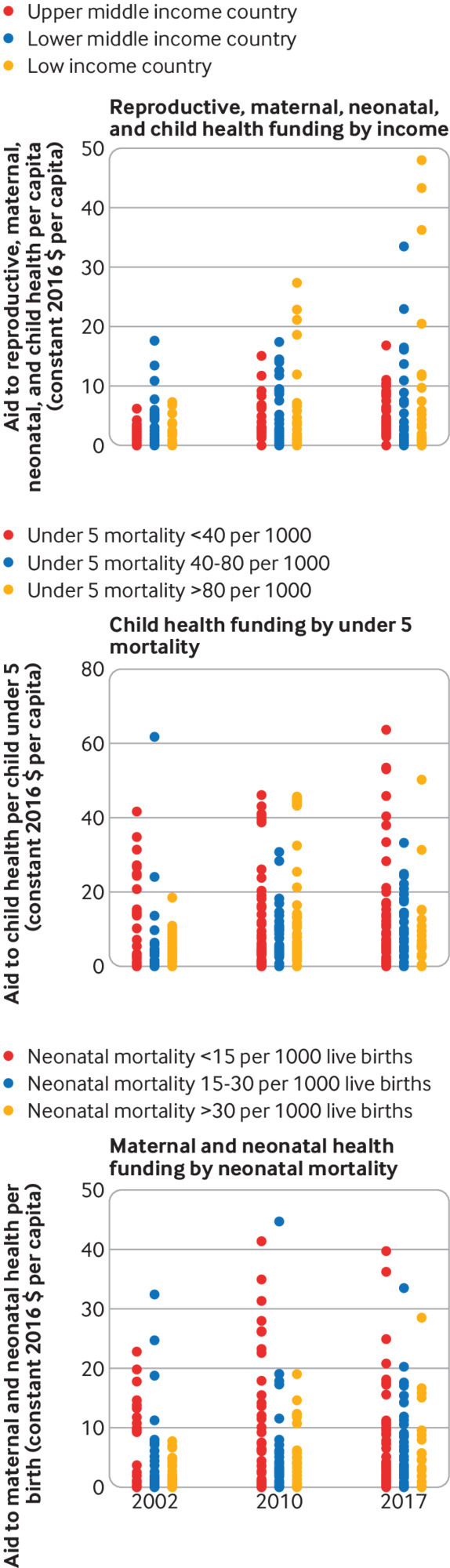
Horizontal equity of donor funds for reproductive, maternal, neonatal and child health (RMNCH) between 2002 and 2017^4^: aid to RMNCH by maternal and neonatal health (top), aid for child health by number of children under 5 (middle), and aid to maternal, and neonatal health per birth (bottom). Countries are grouped respectively by neonatal mortality, under 5 mortality, and country income group to show within group horizontal inequity. Countries were categorised using the World Bank’s income groups based on 2017 data^19^

*Our analysis of Muskoka2 data shows that *the distribution of donor funding is vertically equitable with respect to income, as poorer countries generally receive higher amounts of funding than richer ones, as concentration curves were all above the line of equality (fig 3). From 2002 to 2017, RMNCH funding became increasingly concentrated among the poorest countries; concentration curves moved further from the line of equality (concentration index changed from −0.36 to −0.57). For example, the poorest countries, comprising 10% of the world’s population, received 33% of donor funding for RMNCH in 2002, 42% in 2010, and 47% in 2017. However, we found countries of broadly similar income levels received increasingly different levels of aid for RMNCH between 2002 and 2017 (indicating decreasing horizontal equity) (fig 4).

When using mortality as a measure of need, we found donor funding for child health was concentrated among countries with higher child death rates (above the line of equality in fig 3). This shows vertical equity, but the small decrease in the concentration index between 2002 (−0.25) and 2017 (−0.32) indicates vertical equity improved only marginally. Differences between the levels of donor funding for child health received by countries with similar death rates increased over the same period, indicating aid for child health became less horizontally equitable (fig 4).

In 2002, aid for maternal and newborn health was close to the line of equality (concentration index=−0.06), indicating no correlation with need, but became increasingly concentrated among countries with higher neonatal death rates (indicated by an upward shift of the concentration curve, fig 3). By 2017, the concentration index for aid for maternal and newborn health reached −0.31, almost equal to that of child health funding (web supplement). Differences in funding per birth received by countries with similar neonatal mortality grew between 2002 and 2010, indicating donor funding for maternal and newborn health became less horizontally equitable; this pattern remained similar in 2017 (fig 4).

**Fig 5:**
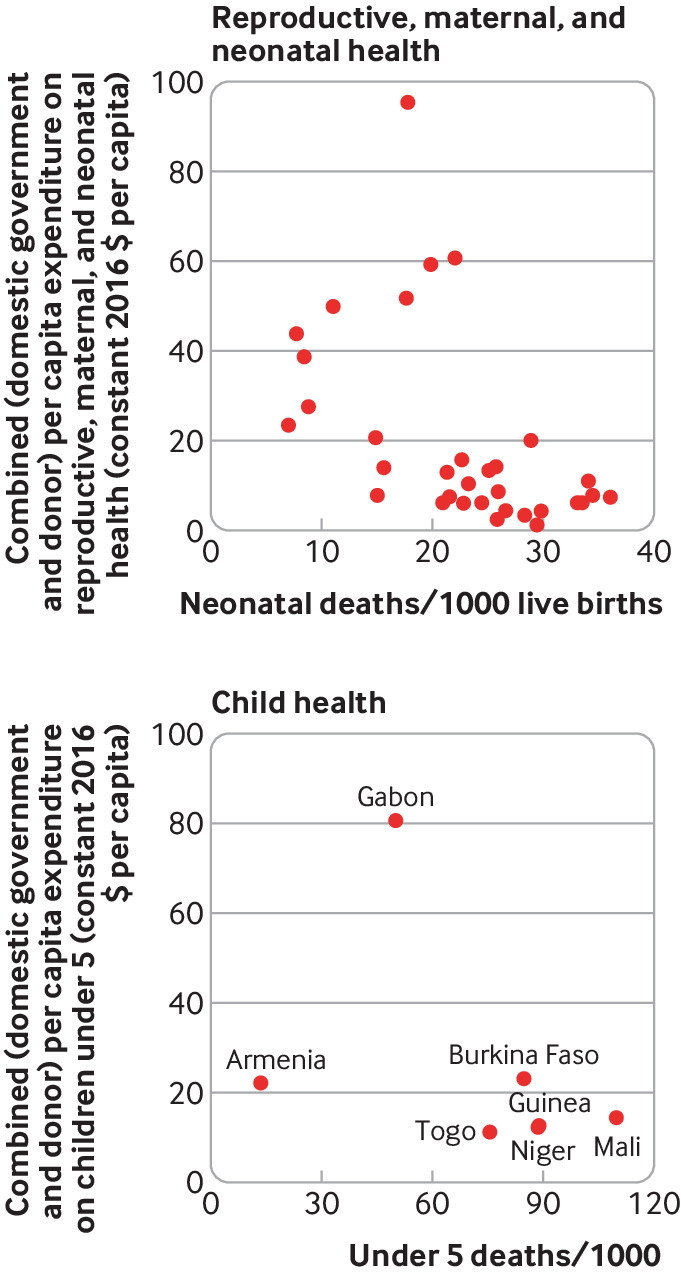
Combined domestic government and donor funding for reproductive, maternal, neonatal, and child health by neonatal and under 5 mortality in 2016 (constant 2016 $PPP per capita)

We used the GHED to assess the distribution of funding from combined domestic government and external sources in relation to country income and health need and found deep inequities (fig 5). Countries with the highest child and maternal mortality received the least child and reproductive health funding, indicating funding is vertically inequitable. Funding was also horizontally inequitable, with substantial variability across countries with similar needs. Guinea, for example, has one of the highest neonatal death rates—over 40 deaths/1000 live births—but received just $6 per capita in combined domestic government and external funding for reproductive health in 2016 (fig 5). For countries with data on family planning in 2016, combined domestic government and external expenditures were higher across three upper middle income countries (mean $2.9 per capita, range: $0.7- $5.2) than nine lower middle income countries ($1.9, $0.2-$6.4) and 13 low income countries ($1.7, $0.1-$6.1), with wide variation within income groups (see web supplement).

Our assessment of trends in equity for RMNCH funding is tentative given the small number of data points available in GHED. For countries with more than one year of data, we examine changes in combined domestic government and external funding between that country’s first and last reported year. For reproductive health, we found that only four of 13 low income countries, nine of 14 lower middle income countries, and two of three upper middle income countries had expenditure increases (web supplement). For family planning, about half of low income (6/12) and lower middle income (3/7) countries, and all three upper middle income countries had expenditure increases (web supplement).

## Priorities and challenges

The increases in donor funding for RMNCH from 2010 to 2017 are encouraging signs of improvement in equity globally. The distribution of aid for RMNCH also became more vertically equitable over this period, in that donors increasingly prioritised lower income countries and those with higher mortality. However, concerns remain regarding the horizontal equity of donor funds, as countries of similar income groups and health needs received increasingly different funding levels. As expected, domestic government expenditure on reproductive health (including maternal health and family planning) and children under 5 tended to be higher in higher income countries, although with substantial variation. Assessment of trends over time was hampered by lack of data but suggests that combined domestic government and external expenditure for RMNCH in low and middle income countries has increased more in the higher income countries than in the poorest countries. This is concerning because it suggests external funds for RMNCH are insufficient to overcome the low levels of domestic government funding in some of the poorest countries, leaving women and children vulnerable to impoverishing expenditure and possibly worse health outcomes.

The global community has placed increasing emphasis on domestic financing,^8^ in part because of concerns that governments may become dependent on external funds, potentially decreasing contributions to areas prioritised by donors. Although donors consider many factors when allocating resources, substantial inequities between countries with the greatest ability to pay and those with the greatest health needs will continue to widen unless governments and donors substantially increase their funding and further improve targeting to those most in need.

Assessing equity of resource flows remains challenging because of data and methodological limitations. Data limitations prevented us from examining out-of-pocket financing at all levels and equity of RMNCH funding within countries. WHO must be commended for its work with countries to collect and share data through the GHED. However, the number of countries reporting funding data for RMNCH priorities is limited, those countries with data report for only a limited number of years, and no data are available on out-of-pocket expenditure for RMNCH, which severely limits any assessment of the equity of financing. While data on total health funding are much more complete, data on funding for other disease or population groups (eg, non-communicable diseases) are also scarce. 

Limitations of the Muskoka2 approach have been extensively discussed elsewhere.^4^ Muskoka2 is applied to data^9^ that include many but not all donors (China’s contributions to aid financing, for example, are not included), and in which aid is categorised by sector and specific subsectoral areas, none of which are specific to child health. The Muskoka2 approach therefore uses various assumptions to disaggregate aid specific to RMNCH from aid for other areas. Although these estimates have been developed through expert opinion and previous analyses of funding distribution,^4^ they are necessarily uncertain.

Improved methods are needed to assess and monitor equity. Consensus is needed on which funds should be considered to benefit RMNCH. Global tracking initiatives such as Muskoka2, for example, consider a proportion of health systems financing to benefit RMNCH, but there is currently no analogous approach for domestic expenditures. Better methods are also necessary to track funding for adolescents, who have different health needs and access to services from other age groups and yet use many of the same services. 

Finally, assessing progress requires a definition of what equitable financing for RMNCH would look like. We have discussed the importance of considering both horizontal and vertical equity, both for “who pays for” and “who benefits” from RMNCH resource flows, and shown the potential to apply this approach both globally and within countries. However, important questions remain. Out-of-pocket financing is widely recognised as the least equitable of financing sources, but there is no agreement on whether donor or domestic government funds are more equitable. In an extreme case, aid funds from the wealthiest donor may be seen as more equitable than funds from poor citizens in the poorest country, but the equity is less clear for funds from a middle income donor. Ultimately these assessments are difficult to make without data on the source of funds, both from donors and from the tax base of recipient countries. 

We recognise taking a global lens to equity may generate debate, particularly within the donor community, which considers many issues beyond equity when allocating funds. We hope to provide an empirical base for debate, and to contribute towards development of more robust methods to assess equity at the global level. More country level studies are also needed to assess the subnational equity of resource flows for RMNCH.

## Conclusions

To achieve the sustainable development goals and universal health coverage, the international community must focus on countries—and populations within them—with highest needs. Despite many limitations of the available data, our analysis suggests that external RMNCH funding favours countries of lower income and higher mortality levels but is not sufficient or sufficiently targeted to overcome the deep inequities in domestic funding. Countries of similar needs continue to receive different funding levels. Although it is vertically equitable that poorer countries receive more aid than better-off countries, combined donor and domestic government funding levels remain inequitable across low and middle income countries. The result is that countries with the lowest income and highest mortality levels are at risk of being left behind in global improvements. To improve equity within and between countries, domestic government expenditure on RMNCH should be increased, although the extent to which governments are able to do so depends on many factors, including national income. To close gaps between countries and increase equity globally, donors need to increase their prioritisation of countries with the highest levels of mortality and the least ability to pay. We call for greater investment in producing and scrutinising data on resource flows for women, children, and adolescents.

## Web extra

Extra material supplied by authors

Details of data available to assess RMNCH funding

## Notes

Contributors and sources: MMA and CP conceived the paper. CP and FF conducted the analysis. MMA and CP wrote the first draft of the manuscript with assistance from all authors. MMA is the guarantor of the article. All authors edited, read, and approved the final version of the text. Sources used are publicly available datasets, survey reports, policy documents and peer reviewed literature. The authors alone are responsible for the views expressed in this publication.

Provenance and peer review: Commissioned; externally peer reviewed.

This article is part of a series proposed by Countdown to 2030 for Women’s, Children’s and Adolescents’ Health and the Partnership for Maternal, Newborn and Child Health (PMNCH) hosted by the World Health Organization and commissioned by *The BMJ*, which peer reviewed, edited, and made the decisions to publish these articles. Open access fees are funded by the Bill and Melinda Gates Foundation and PMNCH.
