# Supplementary material for: Equity of resource flows for reproductive, maternal, newborn, and child health: are those most in need being left behind?
Source: BMJ. 2020 Feb 3;368:m305. doi: 10.1136/bmj.m305 (PMC7461904; doi:10.1136/bmj.m305)
Supplement: Supplementary file 1 — Details of data available to assess RMNCH funding [file marm053357.ww1.pdf]

Supplementary File

Contents

What data are available to assess equity of resource flows for women's, children's, and adolescents' health? 2

Tables 5

Figures 10

References 15

What data are available to assess equity of resource flows for women's, children's, and adolescents' health?

Numerous global financial tracking initiatives allow some assessment of the equity of RMNCH financing between countries health areas, and demographic groups; however, the data gaps are substantial. No initiative has tracked aid for all dimensions of RMNCH either for donor or domestic financing. Those that have tracked financing for one or more dimensions of RMNCH have not used consistent definitions, leading to seemingly similar metrics that are in fact not directly comparable. We review several notable sources of estimates of donor and domestic financing for RMNCH. We restrict our review to initiatives that have generated recent estimates, defined as including data for 2015 or later.

The World Health Organization (WHO) collaborates with countries to produce agreed annual health financing estimates for up to 190 countries each year using the System of Health Accounts 2011[1] (this Supplementary File, Table 1). These estimates, published in the Global Health Expenditure Database (GHED), distinguish the source of financing (i.e. domestic government, external, out-of-pocket), provider, and function of all health expenditure within a country. For some countries, the function of current health expenditure from domestic government and external sources is also disaggregated by beneficiary characteristics,[1] many of which are specific or highly relevant to RMNCH. Age-specific categories indicate the value of health expenditure on children under five; however, these child health data are only available for nine countries and only for the years 2015 and 2016. "Disease"-specific categories (which overlap with the age-specific categories) indicate the value of spending on family planning, reproductive health, immunization programmes, and nutritional deficiencies; however, only 19 countries have data on all 8 of these metrics in 2016, and even fewer countries have data for earlier years. Expenditure estimates are also available for malaria and HIV, which are highly relevant to RMNCH, but these share similar data gaps. The scope for assessing time trends with these metrics is therefore limited, and the metrics themselves do not disaggregate spending on older children or adolescents; for wider aspects of sexual health beyond reproductive health, family planning, and HIV; or out-of-pocket expenditure by households on these areas, which is often substantial. These limitations make it difficult to compare data across years, particularly for specific disease areas.

The Global Fund, GAVI, and the Global Financing Facility (GFF) also collect and analyze domestic health financing data, which they need for their own operations and policies, including on co-financing and transition planning. However, the data from these organizations are only made publicly available in consolidated datasets that cover multiple years and countries over time.

Several initiatives have sought to estimate the value of donor aid for RMNCH in LMICs.[2] These initiatives have made different choices about how to draw boundaries around what to include and what to exclude. These initiatives all exploit the publicly available data provided by government, private, and multilateral donors to the Organisation for Economic Co-operation and Development (OECD). Some initiatives complement these data with additional sources. The OECD's aid activities database distinguishes between humanitarian and health sector aid, which makes it difficult to track aid for health in humanitarian contexts. Humanitarian data are also available from the United Nations Office for the Coordination of Humanitarian Affairs United Nations Office for the Coordination of Humanitarian Affairs' Financial Tracking Service. These data are available for different sectors (including health), but do not allow for easy disaggregation by disease area or population group.

The most recent initiative tracking external expenditure to RMNCH, Muskoka2, tracks aid for RMNCH using a transparent and automated algorithm applied to the OECD's data.[3] It reflects an extensive stakeholder consultation process and is a harmonized approach promoted jointly by the Countdown to 2030 and the Partnership for Maternal, Newborn, and Child Health.[3] The Institute for Health Metrics and Evaluation (IHME) estimates donor and domestic health spending for 195 countries and territories, but only its donor funding estimates – which are based on OECD data and other sources – are disaggregated by health area.[4] These health areas include reproductive and maternal health and child and newborn health. Muskoka2 seeks to reflect the full value of aid directly benefitting RMNCH. whereas IHME's estimates reflect the value of funding targeted towards RMNCH, and thus exclude funding for malaria, HIV, humanitarian aid, and basic health care (which they track separately) from their definition of RMNCH, even where these may directly benefit RMNCH.

Other initiatives have generated estimates of donor or donor and domestic financing for narrower dimensions of RMNCH, notably family planning, nutrition, and adolescent health.[5 6] In 2019, FP2020 reported on domestic government expenditures on family planning for the first time, with validated data from 31 FP2020 focus countries, each covering a single year in 2013, 2014 or 2016.[5] The Kaiser Family Foundation tracks aid for family planning by collecting data directly from the top 10 donors and using the OECD CRS data for other donors; its latest report covers the years 2012-2017, but does not report findings by recipient country.[6]

Several organizations track domestic and donor nutrition financing. The Scaling Up Nutrition (SUN) Movement supports countries to track their "nutrition-relevant budgets" across numerous sectors, including (in order of spending) social protection, health, agriculture, water and sanitation, and education.[7] Their estimates of nutrition-specific and nutrition-

sensitive budget allocations have been generated by 50 countries, but budgets are not spent consistently, so do not reliably indicate resource use. The OECD's dataset includes a "basic nutrition" sub-category within the health sector, which allows analysis of donor disbursements over the period 2002-2017 and is included within the overall Muskoka2 estimates of aid for RMNCH. Research for Development (R4D) tracked aid for specific high-impact nutrition interventions in the OECD's dataset relative to the World Health Assembly investment framework for nutrition,[8] which took a multisectoral perspective, but only produced estimates for the years 2015-17.

Funding for adolescent health is included within funding for many health areas, notably family planning, HIV, and maternal health, but not disaggregated explicitly. Li and colleagues estimated "adolescent-targeted" and "adolescent-inclusive" aid over the period 2003-2015 by analysing the OECD's data.[9] They used a broader definition of "adolescents" (as people aged 10 to 24) than WHO's definition of ages 10 to 19.

Individual countries also have additional financing data, some of which is publicly available and specific to RMNCH. Domestic financing data may be found in budget books, which include data on actual and projected expenditure, typically by sector, but sometimes by disease priority. Some countries also produce public expenditure reviews of the health sector, often including expenditure data on specific priorities, such as maternal health, HIV and malaria.

Tables

Table 1 Available WHO data on RMNCH-related financing indicators

This table shows the number of countries for which data are available in all years for each RMNCH-related financing indicator, as well as current health expenditure.

Source	Indicator	Indicator	1990-2001	2002	2003	2004	2005	2006	2007	2008	2009	2010	2011	2012	2013	2014	2015	2016	2017	2018
**WHO SHA2011**	Domestic general government expenditure on	contraceptive management (family planning)										4	7	10	17	21	20	26		
		HIV/AIDS and sexually transmitted diseases										4	7	14	21	28	30	36		
		immunization programmes										0	0	0	0	0	0	30		
		malaria										4	7	14	21	28	28	32		
		nutritional deficiencies										4	7	14	21	28	29	35		
		reproductive health										4	7	14	21	28	29	35		
		the < 5 year-old population										0	0	0	0	0	7	7		
	External sources of funding on	contraceptive management (family planning)										3	7	12	17	22	24	31		
		HIV/AIDS and sexually										4	7	14	21	27	28	35		

		transmitted diseases																		
		immunization programmes										0	0	0	0	0	0	31		
		malaria										4	7	14	20	25	26	30		
		nutritional deficiencies										4	7	14	20	25	27	33		
		reproductive health										4	7	14	21	27	27	34		
		the < 5 year-old population										0	0	0	0	0	7	7		
	Current Health Expenditure (domestic and external)	Current Health Expenditure (CHE) per Capita	190?	190?	190?	190?	190?	190?	190?	190?	190?	190	190	189	188	188	188	185		
**Muskoka2**	Donor funding for	Reproductive health		138	138	138	138	138	138	138	138	138	138	138	138	138	138	138	138	
		Maternal and newborn health		138	138	138	138	138	138	138	138	138	138	138	138	138	138	138	138	
		Child health		138	138	138	138	138	138	138	138	138	138	138	138	138	138	138	138	
**IHME**	Donor funding for	Reproductive and maternal health	138	138	138	138	138	138	138	138	138	138	138	138	138	138	138	138	138	138
		Child and newborn health	138	138	138	138	138	138	138	138	138	138	138	138	138	138	138	138	138	138
		Malaria	138	138	138	138	138	138	138	138	138	138	138	138	138	138	138	138	138	138
		HIV	138	138	138	138	138	138	138	138	138	138	138	138	138	138	138	138	138	138
		Health system strengthening	138	138	138	138	138	138	138	138	138	138	138	138	138	138	138	138	138	138

**R4D**	Donor funding for	Nutrition															138	138		
**Kaiser Family Foundation**	Donor funding for	Family planning										138	138	138	138	138	138		
**FP2020**	Domestic government expenditure on	Family planning										1	1	5	3		21		

Table 2. Concentration indices

Indicator	Concentration indices		
	**2002**	**2010**	**2017**
**Aid to RMNCH per population**	-0.36	-0.45	-0.57
**Aid to MNH per births**	-0.06	-0.19	-0.31
**Aid to CH per children under 5**	-0.25	-0.34	-0.32

Concentration indices have values between -1 and 1. A value of 0 indicates complete equality (and thus no correlation with need), while -1 and 1 indicates complete inequality.[10] When the concentration curve lies above (below) the line of equality, the concentration index attains negative (positive) values. Data were obtained from applying the Muskoka2 method to the Creditor Reporting System of the Organization for Economic Development and Co-operation.[3]

Table 3. Number and proportion of countries with more than one year of data that experienced increased or decreased expenditure on RMNCH between 2010-16, separated by income group.

		**Govt. spending on family planning**	**Govt. spending on reproductive health**	**External spending on family planning**	**External spending on reproductive health**
Low income countries	**# with increase**	4	8	6	3
	**# with decrease**	8	6	8	11
	**Total**	12	14	14	14
	**% with increase**	**33**	**57**	**43**	**21**
	**% with decrease**	**67**	**43**	**57**	**79**
Lower middle income countries	**# with increase**	5	10	4	7
	**# with decrease**	4	4	5	5
	**Total**	9	14	9	12
	**% with increase**	**56**	**71**	**44**	**58**
	**% with decrease**	**44**	**29**	**56**	**42**
Upper middle income countries	**# with increase**	1	2	2	3
	**# with decrease**	1	2	2	1
	**Total**	2	4	4	4
	**% with increase**	**50**	**50**	**50**	**75**
	**% with decrease**	**50**	**50**	**50**	**25**

Data Source: Data were downloaded from the World Health Organization's Global Health Expenditure Database (GHED). Countries were separated into income groups following the World Bank's income groupings of 2017.

Figures

Figure 1 Domestic government and external expenditure on family planning, 2010-2016

These figures show all available data in WHO's Global Health Expenditure Database (GHED) on domestic general government and external financing on "contraceptive management (family planning)". Expenditure data are available for 36 countries for the years 2010 (n=4) to 2016 (n=26). Data points are only shown in the "combined" graph if both domestic government and external expenditure were reported for that country-year. Data on unmet need for family planning was not consistently available for all countries, and so expenditure levels have not been compared with a metric of health need.

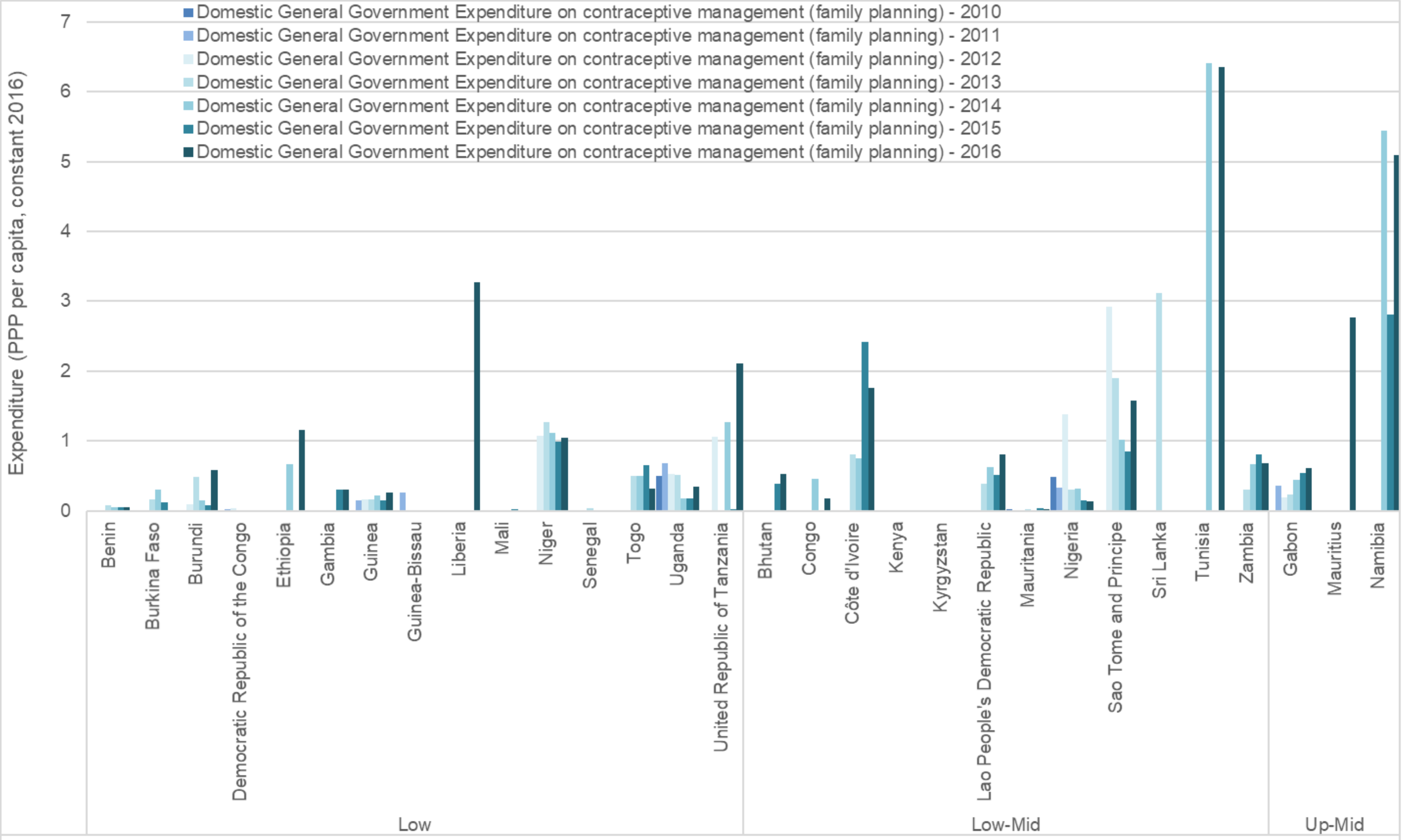
Domestic General Government Expenditure on contraceptive management (family planning) - 2010
Domestic General Government Expenditure on contraceptive management (family planning) - 2011
Domestic General Government Expenditure on contraceptive management (family planning) - 2012
Domestic General Government Expenditure on contraceptive management (family planning) - 2013
Domestic General Government Expenditure on contraceptive management (family planning) - 2014
Domestic General Government Expenditure on contraceptive management (family planning) - 2015
Domestic General Government Expenditure on contraceptive management (family planning) - 2016
Expenditure (PPP per capita, constant 2016)
7
6
5
4
3
2
1
0
Benin
Burkina Faso
Burundi
Democratic Republic of the Congo
Ethiopia
Gambia
Guinea
Guinea-Bissau
Liberia
Mali
Niger
Senegal
Togo
Uganda
United Republic of Tanzania
Bhutan
Congo
Côte d'Ivoire
Kenya
Kyrgyzstan
Lao People's Democratic Republic
Mauritania
Nigeria
Sao Tome and Principe
Sri Lanka
Tunisia
Zambia
Gabon
Mauritius
Namibia
Low
Low-Mid
Up-Mid

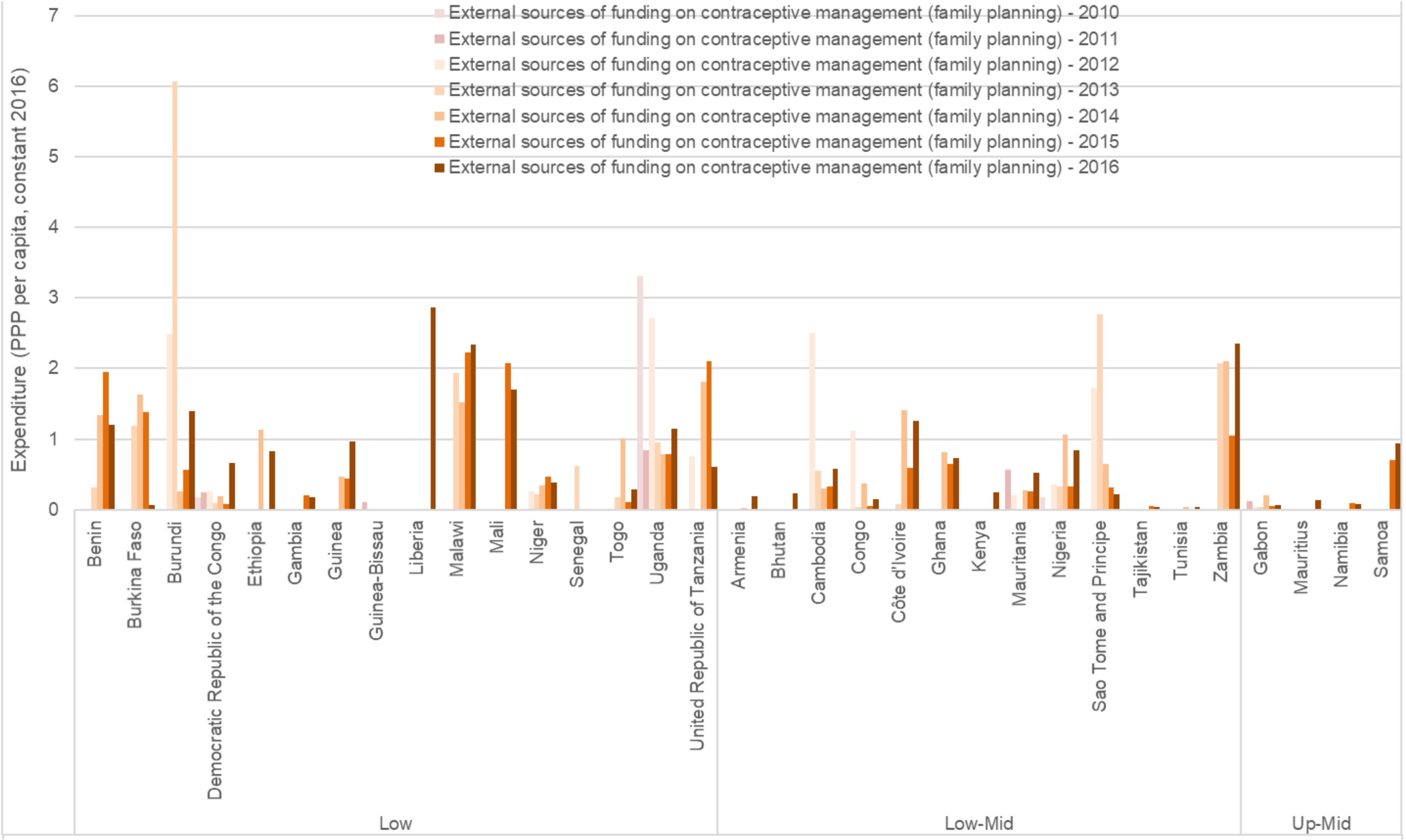

External sources of funding on contraceptive management (family planning) - 2010
External sources of funding on contraceptive management (family planning) - 2011
External sources of funding on contraceptive management (family planning) - 2012
External sources of funding on contraceptive management (family planning) - 2013
External sources of funding on contraceptive management (family planning) - 2014
External sources of funding on contraceptive management (family planning) - 2015
External sources of funding on contraceptive management (family planning) - 2016
Expenditure (PPP per capita, constant 2016)
0
1
2
3
4
5
6
7
Benin
Burkina Faso
Burundi
Democratic Republic of the Congo
Ethiopia
Gambia
Guinea
Guinea-Bissau
Liberia
Malawi
Mali
Niger
Senegal
Togo
Uganda
United Republic of Tanzania
Armenia
Bhutan
Cambodia
Congo
Côte d'Ivoire
Ghana
Kenya
Mauritania
Nigeria
Sao Tome and Principe
Tajikistan
Tunisia
Zambia
Gabon
Mauritius
Namibia
Samoa
Low
Low-Mid
Up-Mid

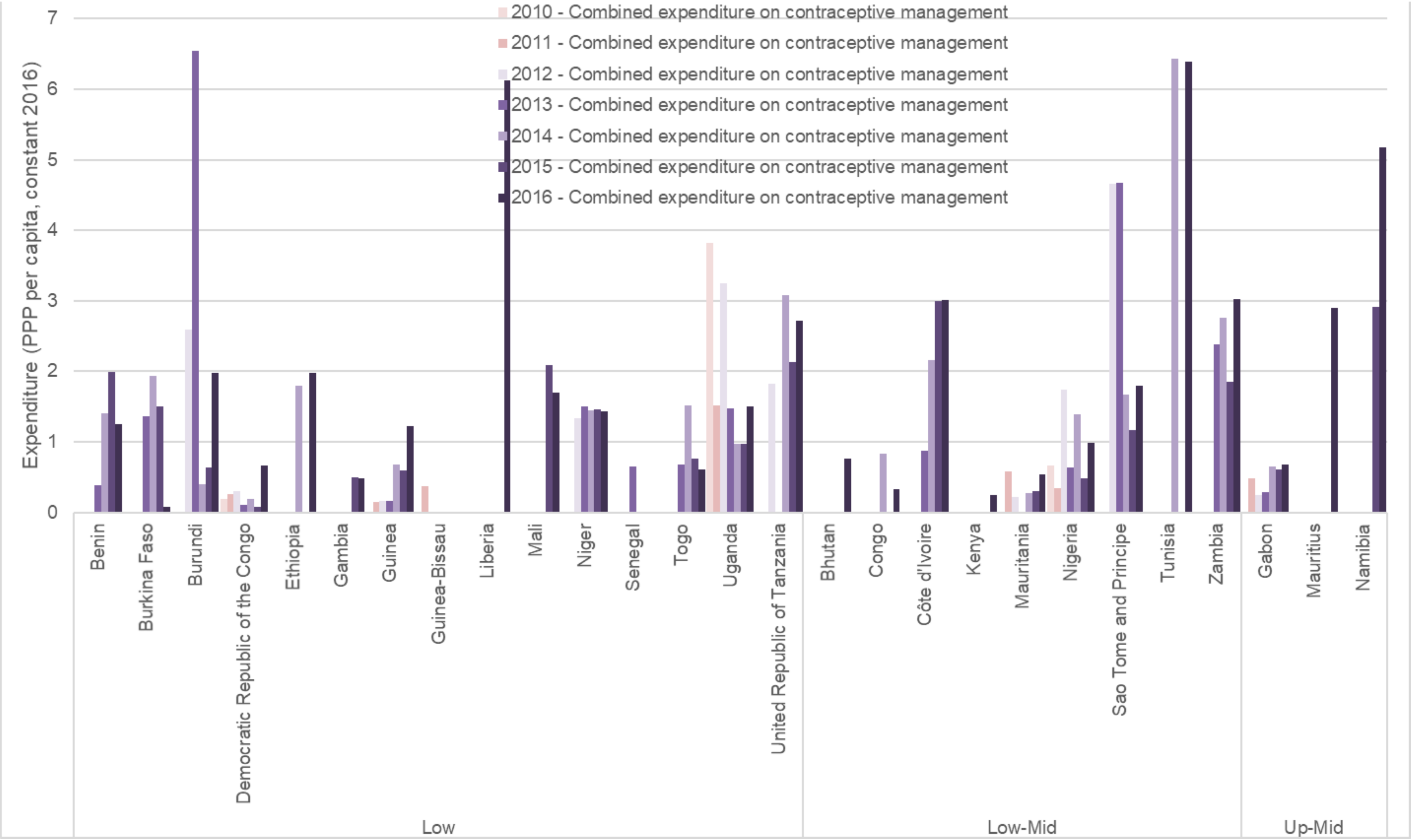

2010 - Combined expenditure on contraceptive management
2011 - Combined expenditure on contraceptive management
2012 - Combined expenditure on contraceptive management
2013 - Combined expenditure on contraceptive management
2014 - Combined expenditure on contraceptive management
2015 - Combined expenditure on contraceptive management
2016 - Combined expenditure on contraceptive management
Expenditure (PPP per capita, constant 2016)
0
1
2
3
4
5
6
7
Benin
Burkina Faso
Burundi
Democratic Republic of the Congo
Ethiopia
Gambia
Guinea
Guinea-Bissau
Liberia
Mali
Niger
Senegal
Togo
Uganda
United Republic of Tanzania
Bhutan
Congo
Côte d'Ivoire
Kenya
Mauritania
Nigeria
Sao Tome and Principe
Tunisia
Zambia
Gabon
Mauritius
Namibia
Low
Low-Mid
Up-Mid

Figure 2 Domestic expenditure on reproductive health, 2010-2016

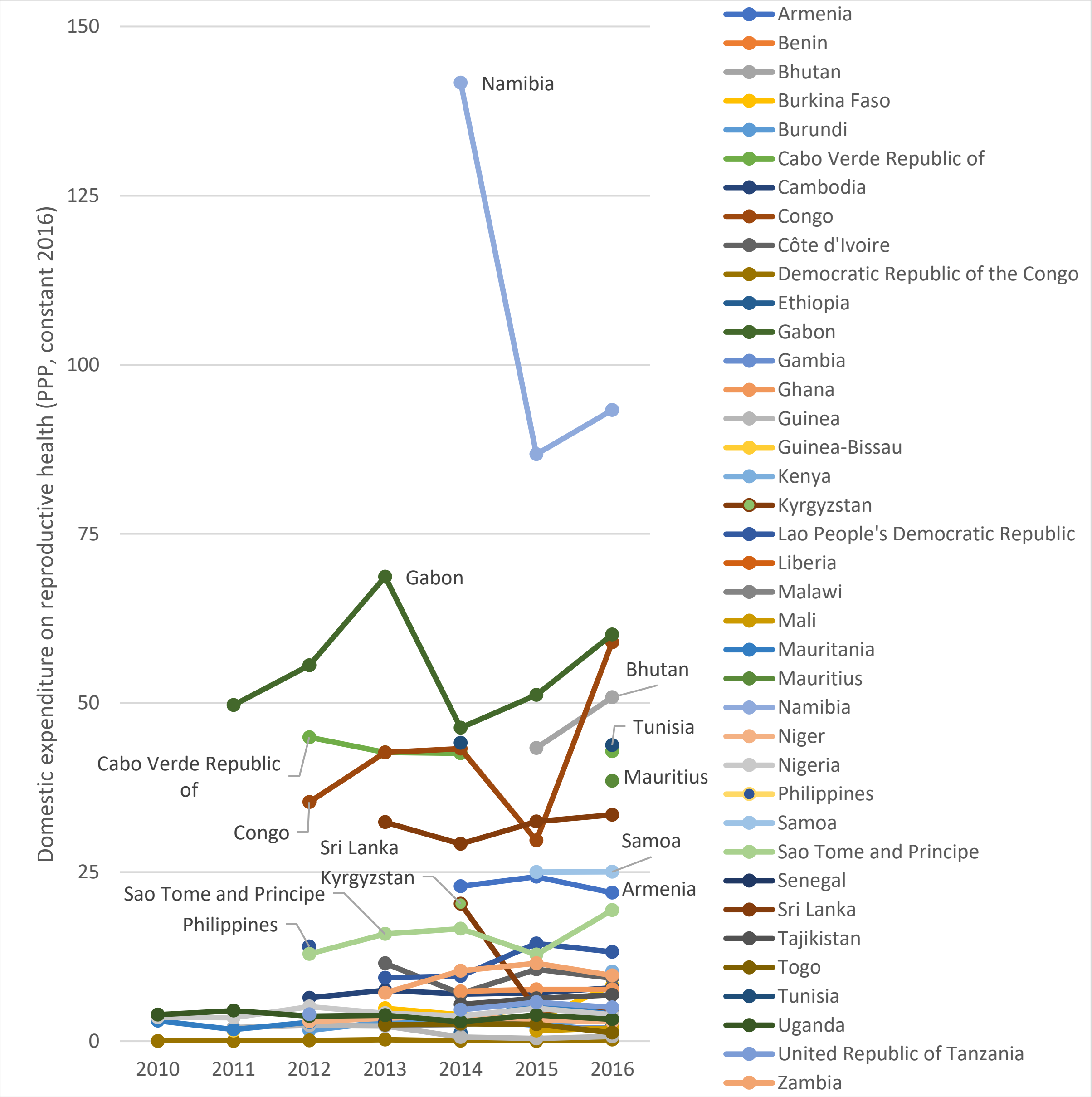

Data source: Data were downloaded from the World Health Organization's Global Health Expenditure Database (GHED).

References

1. OECD, Eurostat, WHO. A System of Health Accounts: OECD Publishing; 2011 [cited 2019 June 19]. Available from: http://apps.who.int/nha/database/DocumentationCentre/Index/en accessed June 19 2019.
2. Pitt C, Grollman C, Martinez-Alvarez M, et al. Tracking aid for global health goals: a systematic comparison of four approaches applied to reproductive, maternal, newborn, and child health. *The Lancet Global Health* 2018;6(8):e859-e74. doi: 10.1016/s2214-109x(18)30276-6
3. Dingle A, Schäferhoff M, Borghi J, et al. Increased aid for reproductive, maternal, newborn and child health: findings from application of the new Muskoka2 method, 2002-2017. *The Lancet Global Health* in press
4. Chang AY, Cowling K, Micah AE, et al. Past, present, and future of global health financing: a review of development assistance, government, out-of-pocket, and other private spending on health for 195 countries, 1995–2050. *The Lancet* 2019;393(10187):2233-60. doi: 10.1016/s0140-6736(19)30841-4
5. Family Planning 2020. Finance: Financing for family planning 2019 [cited 2019 June 20]. Available from: http://progress.familyplanning2020.org/content/finance accessed June 20 2019.
6. Kates J, Wexler A, Lief E. Donor Government Funding for Family Planning in 2017: Henry J Kaiser Family Foundation; 2018 [cited 2019 June 19]. Available from: http://files.kff.org/attachment/Report-Donor-Government-Funding-for-Family-Planning-in-2017 accessed June 19 2019.
7. The Scaling Up Nutrition (SUN) Movement. Annual Progress Report 2018 2018 [cited 2019 June 19]. Available from: https://scalingupnutrition.org/wp-content/themes/elision/pdf/SUNPR-2018/SUN_Report_EN_2018.pdf accessed June 19 2019.
8. Shekar M, Kakietek J, Dayton Eberwein J, et al. An Investment Framework for Nutrition : Reaching the Global Targets for Stunting, Anemia, Breastfeeding, and Wasting, 2017.
9. Li Z, Li M, Patton GC, et al. Global Development Assistance for Adolescent Health From 2003 to 2015. *JAMA Network Open* 2018;1(4) doi: 10.1001/jamanetworkopen.2018.1072
10. O'Donnell O, O'Neill S, Van Ourti T, et al. conindex: Estimation of concentration indices. *The Stata journal* 2016;16(1):112-38.
